# Evaluation of Intraoral Full-Arch Scan versus Conventional Preliminary Impression

**DOI:** 10.3390/jcm12175508

**Published:** 2023-08-24

**Authors:** Kinga Mária Jánosi, Diana Cerghizan, Krisztina Ildikó Mártha, Éva Elekes, Brigitta Szakács, Zoltán Elekes, Alpár Kovács, Andrea Szász, Izabella Mureșan, Liana Georgiana Hănțoiu

**Affiliations:** 1 Faculty of Dental Medicine, George Emil Palade University of Medicine, Pharmacy, Science, and Technology of Târgu Mureș, 38 Gh. Marinescu Str., 540139 Târgu Mureș, Romania; 2 Independent Researcher, 500074 Brașov, Romania; 3 Independent Researcher, 540501 Târgu Mureș, Romania

**Keywords:** intraoral scanning, digital impression, conventional impression, digital dentistry

## Abstract

An accurate impression is vital during prosthodontic rehabilitation. Digital scanning has become an alternative to conventional impressions. This study compares conventional preliminary impression techniques with digital scanning, evaluating the efficiency, treatment comfort, and trueness. Impressions of 28 patients were taken using conventional and digital techniques. The efficiency of both impression techniques was evaluated by measuring the mean working time. A visual analog scale questionnaire (1–10) was used to appreciate the participants’ perceptions of discomfort. Morphometric measurements, which were carried out to determine the differences between the casts, were made on the buccolingual cross sections of teeth 11 and 31 and the distolingual and mesiobuccal cusp tips of each first molar. The total treatment time was 75.5 min for conventional and 12 min for digital impressions. The patients scored a mean discomfort assessment of 6.66 for conventional and 9.03 for digital scanning. No significant differences existed between the examined areas (*p* < 0.05, Wilcoxon and Mann–Whitney tests) of the digital casts obtained by both techniques. The intraoral scan can be considered as an alternative to conventional preliminary impressions for performing study model analysis during orthodontic treatment planning. The digital impression is more comfortable and accepted by the patients than the conventional impression and has a shorter working time.

## 1. Introduction

The dental arch impression is essential to prosthodontists’ and orthodontists’ daily activity. It is used to reproduce the dental arch’s negative to obtain a model for treatment planning, patient communication, or the realization of restorations [1]. The goal of every practitioner is to realize precise impressions, which are a prerequisite for fabricating dental restorations with a proper marginal fit [2]. The accuracy of the casts depends on numerous factors: materials and techniques used; type, size, and rigidity of the impression trays used; application of tray adhesive; shrinkage of the impression materials; type of dental stone; and its compatibility with the different types of impression materials [3,4,5]. Nowadays, the most used impression materials are polyvinyl siloxanes, polyethers, and irreversible hydrocolloids [1]. 

Intraoral scanning was developed in the 1980s and represented an acceptable alternative to conventional impression techniques, providing information for various situations such as diagnosis, orthodontic measurements, restorative dentistry, and implant-supported prosthodontics [4,5,6,7,8]. Improved patient acceptance, comfort, reduced vomiting reflex, reduced distortions, three-dimensional immediate pre-visualization and evaluation of the preparations, cost and time efficiency, more accessible communication with the dental laboratory, and data storage are the benefits of digital scanning [9,10,11]. The intraoral scanners provide direct digitalization by scanning the oral cavity with a camera. The extraoral scanners provide indirect digitalization by scanning a cast resulting from the conventional impression techniques [12,13]. All systems provide only sectional images covering a small area. The scanners’ software converts the data from the scans into STL (standard tessellation language) files, resulting in a three-dimensional image of the jaws. The obtained image’s accuracy depends on the matching algorithm [14]. Although digital scanning shows a rapid and continuous development, it has disadvantages such as difficulty detecting subgingival finish lines of the preparations, inaccuracy in case of bleeding, and moisture near the gingival margins. Artificial reflective surfaces in the oral cavity can also cause errors during scanning [15,16]. A strong advantage of intraoral scanners compared to conventional impressions is the possibility to rescan missing areas or correct mistakes and evaluate the scanned areas by direct visualization on the laptop or computer screen [17]. 

In the case of conventional impressions, mistakes during the recording can sometimes be detected only after pouring the casts; however, corrections must be made by repeating the impression procedure, otherwise the casts will not be accurate. The Trios 3 intraoral scanner’s scanning technology is based on confocal microscopy. Its light source is based on a structured light scanner with infrared light inside [18]. This study compared conventional preliminary impression techniques and digital scanning, evaluating the efficiency, treatment comfort, and trueness of the obtained models.

## 2. Materials and Methods

This comparative study included the upper and lower jaw of 28 patients (14 females and 14 males). The sample size was calculated by using G*Power version 3.1.9.6. software (Franz Faul, Universität Kiel, Kiel, Germany); this size would provide greater than 95% power to detect significant differences, with an effect size of 0.80 at a level of significance of α = 0.05. All the participants were investigated at the Faculty of Dental Medicine of the George Emil Palade University of Medicine, Pharmacy, Science, and Technology of Targu Mures between 10 March 2023 and 20 March 2023. The inclusion criteria were good oral hygiene, fully dentate maxillary and mandibular jaws, an age range of 18–25, and Angle Class I molar relationships with minor malocclusion such as crowding, rotation, or diastema. Patients with systemic health problems and allergies to the materials used, prosthodontic rehabilitation (crowns or bridges), or orthodontic appliances were excluded. 

The clinical trial consisted of digital and conventional preliminary impressions of the dental arches, obtaining the STL files, and performing the dimensional comparison of the digital models. The same medium-experience operator performed the clinical examinations and realized the impressions to obtain comparable testing conditions and reduce or avoid mistakes [19]. 

The study was conducted according to the Declaration of Helsinki and approved by the Ethics Committee of our University (2127/24 February 2023). All the participants provided informed consent in written form. 

### 2.1. Impressions

For digital scanning, the 3Shape Trios 3 intraoral scanner (3Shape A/S, Copenhagen, Denmark) was used, with an LED light source, a scanning accuracy of 6.9 ± 0.9 µm, and a precision of 4.5 ± 0.9 µm. The scanner was calibrated and handled according to the manufacturer’s recommendations [20]. During the scanning procedure, OptraGate Small Refill (Ivoclar Vivadent, Schaan, Lichtenstein) cheek retractors were used to control the accessibility and visibility in the scanning area. The dental arches were gently dried, and a saliva ejector was used to control the saliva. After the warm-up period of the scanner tube (ten minutes), the scanning procedure started at the maxillary arch from the patient’s left side with a scanning path from the occlusal surface of the third molar to the incisors, followed by the lingual and the buccal scan of the dental arches [21,22]. The scanner head was maintained at 0–5 mm from the teeth. It is recommended to wait for about five scanner clicks before continuing the scan to obtain a good starting point. The head of the scanner was moved slowly and gently, and a faster click was heard during the continuous scan. While checking the scanning procedure on the screen, the missing areas were corrected. All the intraoral scans were performed under the same uniform light conditions [23], avoiding the dental chair’s light reflection into the patient’s mouth. For each patient, a single scan was performed.

For the conventional full-arch impressions, the Kromopan (Lascod S.p.a., Sesto Fiorentino, Florence, Italy), a chromatic irreversible hydrocolloid impression material with 168 h of dimensional stability, was used. The color changes will help the practitioner optimize the material’s working and setting time (purple: mixing period; pink: loading the tray; white: setting period). The material was prepared according to the manufacturer’s recommendations. The powder was extracted from the package by using a measuring spoon. For each spoon full of powder, a 1/3 measure of water was added to the mixing bowl and was mixed until the consistency and color were homogenous. A sterile, standard, perforated plastic impression tray with accurate dimensions was used for each impression. Alginate adhesive was used to prevent the displacement of the material during the removal of the tray from the mouth. The patients were asked to rinse their mouth with water to eliminate mucin and decrease the surface tension of the teeth for eliminating air bubbles during impression. The impression tray with the alginate was inserted in the mouth by retracting, with a dental mirror, the lips of the patient on one side and rotating, from the other side, the impression tray into the mouth. After the tray was centered and seated on the dental arch, the pressure was released and the tray was maintained lightly in place to avoid distortions. The setting of the alginate materials starts from the tooth surface to the tray. After thirteen seconds of setting time, the impressions were removed from the mouth, examined for defects under good lighting conditions, rinsed, disinfected, stored in sealed plastic bags, and sent to the laboratory and pored immediately. 

The type IV SheraPremium universal super hard die stone (Shera Material Technology GmbH & Co. KG, Lemförde, Germany) was used to pour the casts. After two hours, the hardness of the stone was 270 MPa, with a setting expansion of 0.10%. The hardness after 24 h was 290 MPa. The working time was 4.5–5.5 min, and the setting time was approximately 30 min. The obtained casts were scanned using a Medit Identica Hybrid 3D laboratory scanner (MEDIT corp., Seoul, Republic of Korea). This scanner has three axes and three color cameras (static part) with high resolution (<7 µm) and a flexible multi-die plate (active part) for the automatic scan of up to eight models at the same time. The scanning time was reduced by 74% by the scanner’s blue light LED scan technology. With the intelligent multi-view scan technology, areas that are difficult to capture (interproximal areas, undercuts) could be scanned safely and quickly. 

The digital data obtained from the scans were converted into STL files compatible with the Exocad software (Rijeka 3.1). 

### 2.2. Comparison of the STL Files

**Figure 1:**
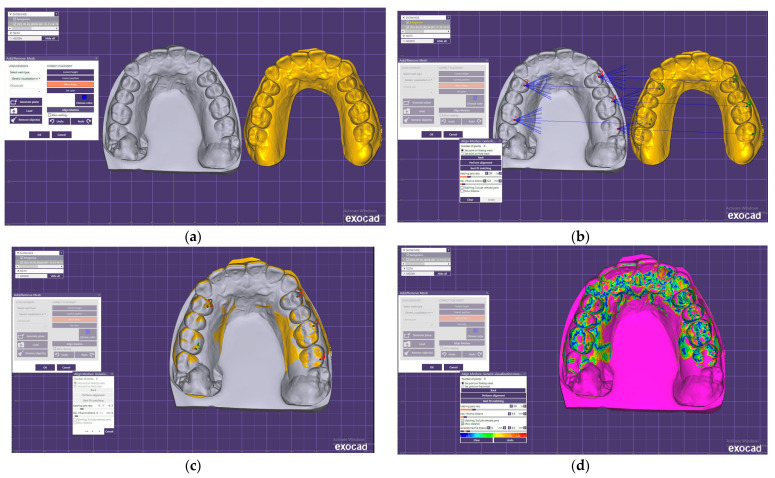
The digital scans in Exocad software: (**a**) study model obtained by indirect scan (gray) and study model obtained by direct scan; (**b**) the reference points for the tripoding procedure; (**c**) superimposed digital models; (**d**) color scale for the differences in the digital scans.

Twenty-eight upper and lower arch impressions were obtained with both techniques. The digital data obtained by indirect and direct scanning were imported into Exocad software (Figure 1a) and superimposed by the tripoding procedure and the best-fit algorithm of the software. The best-fit algorithm is capable of aligning STL files by a set of measured points to match, as closely as possible, that of their counterpart. It can calculate discrepancies between images automatically and make it easier to visualize the discrepancies between the images by color. The disadvantage of the best-fit algorithm is that the deviation may be different from what occurs during the intraoral scanning. For smaller scans (one quadrant) it seems to be suitable, with an acceptable error range [24].

During this study, five reference points were used for a higher superimposition accuracy of the digital models obtained by direct and indirect scans. Two reference points were localized at the palatal cusps of the right first premolars and first molars. Three reference points were located at the buccal cusps of the left first premolars and first and second molars (Figure 1b,c). The differences between the superimposed digital casts were examined using a color scale (Figure 1d). The cold shades of the spectrum indicated minor differences between the two models, while the warm colors showed increasingly significant differences between the digital scans.

**Figure 2:**
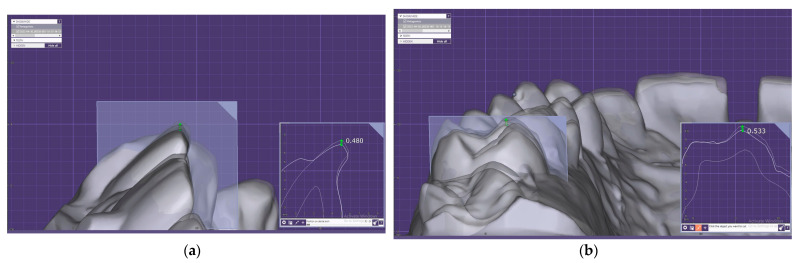
The morphometric measurements in Exocad software: (**a**) differences in the cross section of tooth 11; (**b**) differences in the cross section of tooth 16.

The morphometric differences were evaluated by measurements of the buccolingual cross sections of teeth 11 and 31 and of the distolingual and mesiobuccal cusp tips of teeth 16, 26, 36, and 46; the FDI dental notation system was used (Figure 2).

### 2.3. Time Efficiency, Patient Point of View

A second operator recorded the duration of each conventional impression or intraoral scan in seconds, and for statistical interpretation, the results were converted in minutes. The mean of total treatment time was calculated for both techniques. For conventional preliminary impressions, the working time was recorded from the moment of preparing the impression material until the scanning of the study model with the laboratory scanner for the upper and lower arches was performed. For the digital impressions, the time was recorded after the complete warm-up of the scanner tip, from the beginning of the intraoral scan until the data were imported into the software. 

The patients were asked to answer a visual analog scale questionnaire (1—lowest score, 10—highest score), similar to that of Yuzbasioglu et al. [3], to score their overall discomfort and perception of the effectiveness of the methods used.

### 2.4. Statistical Analysis

The statistical analysis was performed by using GraphPad Prism 9 for macOS version 9.5.1 software. The outlier analysis and exclusion were performed using the ROUT method. The statistical significance was set at *p* < 0.05. The mean (M), median (Me), and sta ndard deviation (SD) were calculated. The Shapiro–Wilk test was used to check the distribution. Wilcoxon and Mann–Whitney tests were also used.

Null hypothesis:

- There are no significant differences between the conventional preliminary impression technique and intraoral scan trueness.
- The digital impression is more comfortable and less time-consuming than the conventional preliminary impression.

## 3. Results

The descriptive statistics of the morphometric measurements obtained for both impression techniques are presented in Table 1.

To observe that there is no statistical difference between the two impression methods, the standard acceptable value was set at zero. The results of the Wilcoxon test revealed that the difference between the two impression methods is statistically significant (*p* < 0.0001). The Mann–Whitney test was applied to determine if there were differences between the values obtained at the lower and upper arch. The results are presented in Table 2.

No statistically significant differences were found regarding the scanned area, except for the frontal and lateral left inferior arch. The results obtained by comparing the values on the same dental arch are presented in Table 3.

The mean value of the working time for the conventional impression of both arches was 75.50, and for the digital impression it was 12.00 (*p* < 0.0001, Mann–Whitney test).

On a 1–10 visual analogue scale, the patients scored the discomfort and effectiveness of the impression techniques used. The obtained data and results of the Mann–Whitney test are presented in Table 4.

## 4. Discussion

The present study investigated the trueness of casts resulting from digital and conventional full-arch impressions in fully dentate young patients. The trueness of the impression represents the difference in the obtained geometry compared to the original, while the precision of the impression represents the differences between repeated impressions [25]. According to Sanda et al., trueness indicates the degree to which the digital scan reproduces the analog cast or dental arch and precision shows the degree to which the digital models obtained by repeated scans of a model or dental arch correspond with each other [26]. According to the International Standard Organization (ISO) definition from 1994 [27], both factors must be considered when the accuracy of the impressions is examined. Digital impressions are becoming increasingly common due to their comparable accuracy to conventional impressions [28]. In this study, precision between the conventional and intraoral scans was not evaluated, only trueness. To evaluate the trueness of the impressions, gold-standard data must be used as the true value. This true value can be obtained by coordinate measuring machines, industrial 3D scanners, or dental laboratory scanners [26]. The extraoral scan was considered a reference in this study because the stone casts resulting from conventional impressions are still commonly used in orthodontics. The accuracy of industrial scanners ranges from 1 to 10 µm; the accuracy of laboratory scanners ranges from 2 to 10 µm. Therefore, the accuracy of a digital model obtained by laboratory scanners is comparable to that of an industrial 3D scanner [29], with interpretable results. 

Before each digital impression, the intraoral scanner was calibrated according to the manufacturer’s recommendations [19] to avoid possible mistakes. The recommended scanning path was used during the intraoral scans: from occlusal to palatal towards buccal. According to Müller et al., this scanning strategy provides the highest precision and trueness in full-arch scans and minimizes the mistakes and inaccuracies of the final working models [30]. According to Schirmer and Wiltshire [31], measurement differences of less than 0.20 mm for orthodontic study models were clinically acceptable. This reference value was determined to be higher (about 0.30 mm) by Hirogaki et al. [32]. Our results follow those obtained by Hirogaki et al. Bell et al. [33] stated that a 0.27 mm difference is not clinically significant. However, in the case of orthodontic appliances, the 3D printing procedure can increase the differences, leading to a higher number of errors. Our results are acceptable in the case of study casts. No significant differences were found between the values of the morphometric measurements for the upper and lower arches. The average difference between the values obtained with the two impression techniques was 317.8555 microns. The newest intraoral scanners and their more precise registration techniques can lead to better results and/or working models. However, in the case of orthodontic working models, these values can generate differences and misfits of the orthodontic appliances. Several factors can influence the results. The scanners’ different technologies (light, laser, or contact) do not affect the scanner’s overall reliability but the scanning technique. The presence of blood, saliva, or humidity in the scanned area, limited mouth opening, tongue movements, and the patient’s movements can determine inaccurate scans [34,35]. In our study, the main differences were obtained in the case of molars for both arches, probably because of their bigger surface and reduced visibility or access, which can lead to a higher probability of errors. Several studies have reported comparable or even higher accuracy for intraoral scans compared with conventional impressions for short-span fixed prosthodontic works up to a quadrant [36,37,38,39,40]. Other studies demonstrated that the transfer accuracy for full-arch scans was higher in the case of conventional impression techniques when precise impression materials were used (polyvinyl siloxane, polyether, vinylsiloxanether, directly scannable vinylsiloxanethers [12,40,41]), and the casts were scanned with an extraoral scanner [13]. Our findings are like those obtained by Ender et al. in their study [42]. The digital impressions resulted in more accurate digital models than conventional ones [37]. The lower accuracy of conventional alginate impressions was related to the impression material, impression technique, cast pouring, and stone expansion [43,44,45]. The alginate is the least accurate impression material, as demonstrated by Bud et al. in their study [46]. Intraoral scanning has limitations in detecting subgingival finish lines on prepared teeth or in case of bleeding [47]. 

The participants in this study were young patients without exposed root surfaces, undercuts, edentulous spaces, or prosthodontic works, which allowed an easier scan and conventional impression. The examined arches were integral, without deep margins or bleeding areas, and the models obtained were study models for orthodontic treatment planning. The digital impression technique was more effective regarding working time and more comfortable; it was preferred by the patients, with a meaningful assessment of 6.66 for the conventional impressions and 9.03 for the digital impressions. These results are supported by many current studies [11,48,49,50] but contrast with that of Gründheid et al. [51]. According to that study, conventional alginate impression techniques were preferred by patients because of the dimensions of the scanner’s tip. Siquera et al. considered that intraoral scanning procedures can improve the patient experience regarding preference and comfort during impressions [17]. Our results demonstrated that the intraoral scan was less time-consuming than the conventional preliminary impression. The total working time was 75.5 min for conventional and 12 min for digital impressions. These findings are supported by other studies [1,10,49,52,53]. Only a few studies reported a reduced working time for conventional impressions [54,55]. Wismeijer et al. [56] reported a significantly higher overall preference of the patients for using the digital impression technique. However, the perception of the patients regarding the working time of the digital impressions was more negative than in the case of the conventional impressions. 

The operator’s experience positively influences the intraoral scanning time and accuracy. The beginners obtain a scan with a higher number of images and a longer scanning time than medium- and high-experience operators [19]. The operator’s experience can influence the accuracy of both digital and conventional impressions. In our study, the conventional and digital impressions were performed by the same medium-experience person.

The scan size influences the accuracy of the obtained images. Several studies demonstrated the higher accuracy of the smaller scan areas compared to full-arch scans [19]. A noticeable clinical benefit of using intraoral scanners for impressions is reducing the risk of cross infections [57]. Conventional impression materials can suffer dimensional or surface modifications following immersion in different disinfectant solutions [58]. By using conventional techniques, the laboratory team is exposed to different infectious microbial agents, increasing the risk of cross contamination. With a fully digital workflow, the infection risk can be limited to the direct contact of the patient with the scanner’s tip and the dentist. The infection risk can be reduced considerably by using adequate surface disinfectants and sterilization protocols for the scanner tips [57].

The limitations of the present study were: the use of a single impression material and a single intraoral scanner; only tridimensional superimposition of the cast being analyzed; certain areas with or without deviations that could remain unobserved and quantified; and the precision of the impressions not being examined. Other materials and new advanced intraoral scanners, with their more complex workflows, could lead to different, better results. The lack of standardization of the landmarks used to perform the measurements does not allow an accurate assessment of the precise accuracy evaluation. For an accurate assessment of the accuracy, more clinical trials are needed using more impression materials and more intraoral scanners.

## 5. Conclusions

Based on the findings of this study, the following conclusions were drawn:

- The intraoral scan can be considered as an alternative to the conventional preliminary impression for performing study model analysis during orthodontic treatment planning.
- The digital impression is more comfortable and accepted by the patients than the conventional impression and has a shorter working time.
- The performance of the impression techniques used can be corrected with experience and good clinical skills.

## Figures and Tables

**Table 1:** The morphometric differences between the models obtained by conventional and digital techniques at the reference teeth for the upper and lower arches—descriptive statistics.

	Upper Arch (mm)			Lower Arch (mm)		
	11	16	26	31	36	46
**Minimum**	0.1700	0.1040	0.002000	0.1050	0.1320	0.000
**25% Percentile**	0.1785	0.1670	0.2040	0.1673	0.1990	0.1320
**Median**	0.2290	0.2310	0.2675	0.2090	0.2390	0.2530
**75% Percentile**	0.2980	0.3850	0.3480	0.2873	0.4530	0.3880
**Maximum**	0.3310	0.4970	0.6200	0.3460	0.6300	0.4410
**Range**	0.1610	0.3930	0.6180	0.2410	0.4980	0.4410
**Mean**	0.2355	0.2770	0.2581	0.2155	0.3171	0.2426
**Std. Deviation**	0.05577	0.1265	0.1552	0.07027	0.1560	0.1390
**Std. Error of Mean**	0.01054	0.02390	0.02932	0.01328	0.02948	0.02627
**Lower 95% CI of Mean**	0.2139	0.2279	0.1980	0.1883	0.2566	0.1887
**Upper 95% CI of Mean**	0.2571	0.3260	0.3183	0.2428	0.3776	0.2965

**Table 2:** The differences between lower and upper arch.

	Mann–Whitney	Difference	*p*-Value
**11 vs. 31**	301.5	0.02000	0.1394
**16 vs. 46**	338	−0.02200	0.3808
**26 vs. 36**	364.5	0.02850	0.6571

**Table 3:** The differences regarding the values on the same dental arch.

	Mann–Whitney U	Difference	*p*-Value
**11 vs. 16**	353	−0.00200	0.5279
**11 vs. 26**	302	−0.03850	0.1418
**16 vs. 26**	378	−0.03650	0.8229
**31 vs. 46**	350	−0.04400	0.4963
**31 vs. 36**	236	−0.03000	0.0098 **
**36 vs. 46**	295	−0.01400	0.1129

** very significant.

**Table 4:** Patient perception of the impression techniques.

	Conventional Impression	Digital Impression	*p*-Value
**Minimum**	4.000	8.000	<0.0001 ****
**Median**	6.750	9.000	
**Maximum**	8.500	10.00	
**Mean**	6.500	9.018	
**Std. Deviation**	1.333	0.7756	
**Std. Error of Mean**	0.2520	0.1466	
**Lower 95% CI**	5.983	8.717	
**Upper 95% CI**	7.017	9.319	

**** extremely significant.

## Data Availability

The data analyzed in this article are available from the first author on request.
